# Fault Diagnosis of Gearboxes Using Nonlinearity and Determinism by Generalized Hurst Exponents of Shuffle and Surrogate Data

**DOI:** 10.3390/e20050364

**Published:** 2018-05-14

**Authors:** Chunhong Dou, Xueye Wei, Jinshan Lin

**Affiliations:** 1 School of Electronic and Information Engineering, Beijing Jiaotong University, No. 3 Shangyuancun, Haidian District, Beijing 100044, China; 2 School of Mechatronics and Vehicle Engineering, Weifang University, No. 5147 Dong Feng Dong Street, Weifang 261061, China; jslinmec@hotmail.com or

**Keywords:** fault diagnosis, gearbox, nonlinearity, determinism, generalized Hurst exponent

## Abstract

Vibrations of defective gearboxes show great complexities. Therefore, dynamics and noise levels of vibrations of gearboxes vary with operation of gearboxes. As a result, nonlinearity and determinism of data can serve to describe running conditions of gearboxes. However, measuring of nonlinearity and determinism of data is challenging. This paper defines a two-dimensional measure for simultaneously quantifying nonlinearity and determinism of data by comparing generalized Hurst exponents of original, shuffle and surrogate data. Afterwards, this paper proposes a novel method for fault diagnosis of gearboxes using the two-dimensional measure. Robustness of the proposed method was validated numerically by analyzing simulative signals with different noise levels. Moreover, the performance of the proposed method was benchmarked against Approximate Entropy, Sample Entropy, Permutation Entropy and Delay Vector Variance by conducting two independent gearbox experiments. The results show that the proposed method achieves superiority over the others in fault diagnosis of gearboxes.

## 1. Introduction

Fault diagnosis of machines is of great importance for ensuring their safe operation [1,2,3]. In recent decades, many methods, such as vibration signal analysis, acoustic signal analysis and artificial intelligence, have been put forward for fault diagnosis of machines [4,5,6,7,8,9,10,11]. In this sense, fault diagnosis of machines comes into the focus of intensive research because of its considerable potential application [12,13,14]. 

As a typical mechanical part, a gearbox illustrates wide use in industrial fields. Owing to instability of working environments, vibrations of a gearbox usually demonstrate considerable complexities [15,16,17]. On the one hand, a gearbox displays different dynamical behavior under different running conditions [18,19,20]. On the other hand, noise levels of vibrations of a gearbox may vary with degeneration of performance of gearboxes [21,22,23]. In this respect, nonlinearity and determinism of vibration data of a gearbox can seemingly be exploited to characterize running conditions of a gearbox, by representing dynamics and determinism of data, respectively. Nevertheless, there is difficulty in measuring nonlinearity and determinism of vibration data. Currently, some entropy-based methods, for instance, Approximate Entropy, Sample Entropy and Permutation Entropy, have been put forward to gain insight into nature of complex data [24,25,26]. Unfortunately, these entropy-based methods often miss the essence of complex data [25,26]. Recently, delay vector variance (DVV) has been devised for investigating complex data [27]. In principle, DVV can measure nonlinearity of complex data to an extent and gives a poor performance in exposing determinism of data. As a result, DVV frequently shows few capabilities for discovering nature of complex data. Consequently, there is a requirement for finding a different path for quantification of nonlinearity and determinism of data. 

Dynamics of a defective gearbox usually present themselves as multifractality [19,20,28,29]. The generalized Hurst exponent can convey multifractality of complex data, abstracted by multifractal detrended fluctuation analysis (MFDFA) [30,31]. In addition, some transformations of data are essential for delving into data. In this paper, shuffle and surrogate procedures are used to gain insight into nature of data [32]. The shuffle procedure can destroy correlations of original data while maintaining the broad probability density distribution of original data [32]. By contrast, the surrogate procedure can destroy broad probability density distribution of original data while maintaining the correlations of original data [32]. Thus, by comparing generalized Hurst exponents of shuffle, surrogate and original data, nonlinearity and determinism of data can be determined. Specifically, an average of differences between generalized Hurst exponents of the surrogate data and of the original data is used as a measure for nonlinearity of data. In addition, an average of differences between generalized Hurst exponents of the shuffle data and of the original data is used as a measure for determinism of data. Consequently, this paper develops a pair of measures for simultaneously quantifying nonlinearity and determinism of complex data. Following this, a novel method for fault diagnosis of gearboxes is proposed based on the two-dimensional measure. In addition, the feasibility of the proposed method was confirmed numerically and experimentally. 

The remainder of this paper is structured as follows. Section 2 formulates MFDFA, elucidates nature of the generalized Hurst exponent, explains the shuffle and the surrogate procedures and proposes the novel method for fault diagnosis of gearboxes. In Section 3, robustness of the proposed method in different signal-noise-ratio (SNR) conditions is validated numerically. In Section 4, the performance of the proposed method is benchmarked against Approximate Entropy, Sample Entropy, Permutation Entropy and DVV by conducting two independent gearbox experiments. Section 5 opens a discussion. Finally, Section 6 concludes this paper. 

## 2. The Proposed Method for Fault Diagnosis of Gearboxes

### 2.1. Generalized Hurst Exponents

The generalized Hurst exponent can be estimated by MFDFA. The execution of MFDFA for a series $x_{k}$ of length N comprises the next five steps [30]:

(1) Construct a cumulative-difference series Y(i) as follows.
  (1) $$\begin{array}{l} Y\left(i\right)=\sum_{k=1}^{i}\left[x_{k}-\langle x\rangle \right]\\ \langle x\rangle =\frac{1}{N}\sum_{k=1}^{N}x_{k} \end{array}$$
(2) Split Y(i) into $N_{s}=\mathrm{int}\left(N/s\right)$ non-overlapping segments, each with the same length s. To make full use of these data, the same procedure is carried out again in reverse order. Accordingly, altogether $2N_{s}$ data segments are obtained.
(3) Apply the least-square algorithm to fit a local trend of each of the $2N_{s}$ segments. Define the variance as
  (2) $$F^{2}\left(v,s\right)=\frac{1}{s}\sum_{i=1}^{s}{\left\{Y\left[\left(v-1\right)s+i\right]-y_{v}\left(i\right)\right\}}^{2}$$
  for the *v*th segment, $v=1,\ldots ,N_{s}$, and as
  (3) $$F^{2}\left(v,s\right)=\frac{1}{s}\sum_{i=1}^{s}{\left\{Y\left[N-\left(v-N_{s}\right)s+i\right]-y_{v}\left(i\right)\right\}}^{2}$$
  for the *v*th segment, $v=N_{s}+1,\ldots ,2N_{s}$. Here, $y_{v}\left(i\right)$ stands for the fitted polynomial trend in the *v*th segment.
(4) Acquire the *q*th-order fluctuation function $F_{q}\left(s\right)$ by averaging all of the $2N_{s}$ segments as follows.
  (4) $$F_{q}\left(s\right)={\left\{\frac{1}{2N_{s}}\sum_{v=1}^{2N_{s}}{\left[F^{2}\left(v,s\right)\right]}^{q/2}\right\}}^{1/q}$$
  Here, the variable q is a non-zero real number. Adjust the time scale s and repetitively carry out Steps (2)–(4). Consequently, the fluctuation $F_{q}\left(s\right)$ can present itself as a function of q and s.
(5) Establish a power-law relation between $F_{q}\left(s\right)$ and s for different q:
  (5) $$F_{q}\left(s\right)\sim s^{H\left(q\right)}$$
  For q=0,
  (6) $$F_{0}\left(s\right)=\exp\left\{\frac{1}{4N_{s}}\sum_{v=1}^{2N_{s}}\ln\left[F^{2}\left(v,s\right)\right]\right\}\sim s^{H\left(0\right)}$$
  Here, H(q) indicates the generalized Hurst exponent of the series.

### 2.2. Nature of the Generalized Hurst Exponent

With the generalized Hurst exponent H(q), the standard scaling exponent τ(q) can be determined as follows [30].

(7) τ(q)=qH(q)−1

Afterwards, by the Legendre transform, a set of multifractal parameters, consisting of the singularity exponent α and the multifractal spectrum f(α), can be deduced as follows [30].

(8) $$\{\begin{array}{c} \alpha =\tau^{\prime }(q)=H(q)+qH^{\prime }(q)\\ f(\alpha )=q\alpha -\tau (q)=q\left[\alpha -H(q)\right]+1 \end{array}$$

Accordingly, the generalized Hurst exponent expresses essence of multifractality buried in complex data. 

### 2.3. Shuffle and Surrogate Procedures

The shuffle procedure can randomize the sequence of original data. In doing so, the shuffled data lose intrinsic long-range correlations of the original data but inherit the broad PDF of the original data [32].

The surrogate procedure of a series x(t) comprises the following steps [32].

(1) Obtain the phase of x(t) by performing DFT for x(t).
(2) Acquire the surrogate frequency-domain data by substituting a set of pseudo independent and identically distributed quantities in a range of −π and π for the phase obtained previously.
(3) Get the surrogate data by performing inverse DFT for the surrogate frequency-domain data calculated above.

### 2.4. Determination of Nonlinearity and Determinism of Data

According to Equation (5), the deviation of the generalized Hurst exponents of the shuffle and the surrogate data from those of the original data can be estimated as follows [32].

(9) $$F_{q}\left(s\right)/F_{q}^{\mathrm{shuf}}\left(s\right)\sim s^{H\left(q\right)}/s^{H_{\mathrm{shuf}}\left(q\right)}=s^{H\left(q\right)-H_{\mathrm{shuf}}\left(q\right)}=s^{H_{\mathrm{corr}}\left(q\right)}$$

(10) $$F_{q}\left(s\right)/F_{q}^{\mathrm{surr}}\left(s\right)\sim s^{H\left(q\right)}/s^{H_{\mathrm{surr}}\left(q\right)}=s^{H\left(q\right)-H_{\mathrm{surr}}\left(q\right)}=s^{H_{\mathrm{PDF}}\left(q\right)}$$

Here, $H_{\mathrm{shuf}}\left(q\right)$ and $H_{\mathrm{surr}}\left(q\right)$ stand for the generalized Hurst exponents of the shuffle and the surrogate data, respectively. In addition, $H_{\mathrm{corr}}\left(q\right)$ and $H_{\mathrm{PDF}}\left(q\right)$ represent the generalized Hurst exponents of the data only containing long-range correlations and of the data only containing the broad PDF, respectively. Consequently, $H_{\mathrm{corr}}\left(q\right)$ and $H_{\mathrm{PDF}}\left(q\right)$ reveal determinism and nonlinearity of the original data, respectively. Thus, an average of $H_{\mathrm{corr}}\left(q\right)$ can be used as a measure for determinism of the original data and an average of $H_{\mathrm{PDF}}\left(q\right)$ as a measure for nonlinearity of the original data. 

### 2.5. The Proposed Method for Fault Diagnosis of Gearboxes

**Figure 1:**
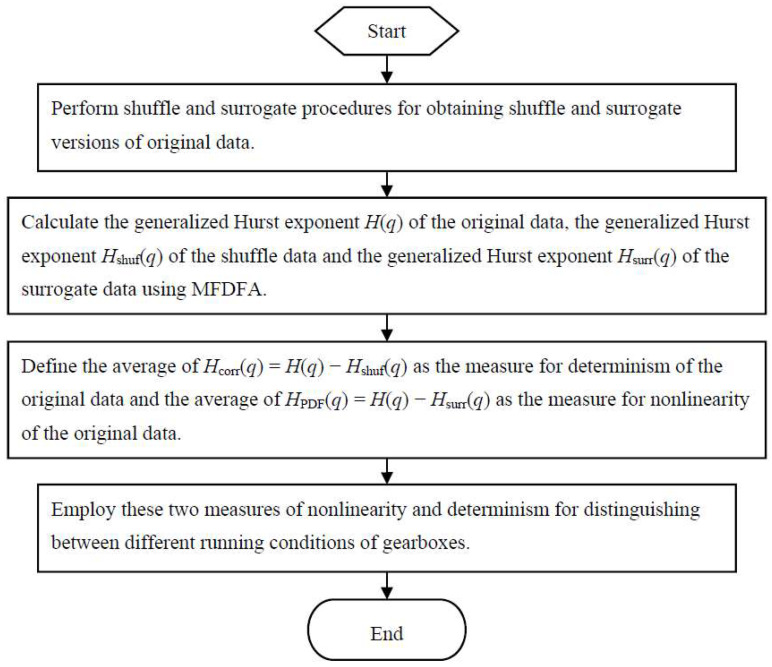
A flowchart of the proposed method.

In this subsection, a novel method for fault diagnosis of gearboxes is proposed using nonlinearity and determinism by generalized Hurst exponents of shuffle and surrogate data. A flowchart of the proposed method is described in Figure 1. 

## 3. Validation of Robustness of the Proposed Method in Different SNR Conditions

In this section, robustness of the proposed method is validated by examining simulative signals of different SNRs. To this end, four types of signal in different SNR conditions were constructed. Each type of signal was composed of ten pieces of data, each piece with a sample frequency of 1000 Hz and a size of 10,000. As a result, ten groups of signals were obtained, each labeled as $\left(x_{0}^{j},x_{1}^{j},x_{2}^{j},wgn^{j}\right),j=1,2,\cdots ,10$. Here, $x_{0}^{j}$ stands for the *j*th pure amplitude–modulation (AM) signal, $x_{1}^{j}$ for the *j*th noisy signal with SNR 10 dB by adding noise to $x_{0}^{j}$, $x_{2}^{j}$ for the *j*th noisy signal with SNR 0 dB by adding noise to $x_{0}^{j}$ and $wgn^{j}$ for the *j*th white Gaussian noise of length 10,000, respectively. The *j*th pure AM signal $x_{0}^{j}$ is expressed as follows.

(11) $$x_{0}^{j}=\cos\left(20\pi t\right)\cos\left(2000\pi t+\pi /6\right)$$

**Figure 2:**
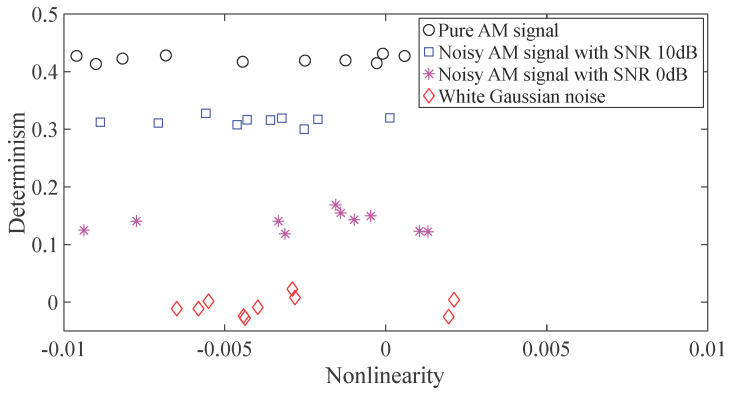
Quantification of nonlinearity and determinism of four different types of signal in different SNR conditions.

Here, $t=\left(0,1,\cdots ,N-1\right)/f_{s},\;f_{s}=1000,\;N=10000$. Next, the proposed method was used to quantify nonlinearity and determination of these four types of signal and the results are displayed in Figure 2. As displayed in Figure 2, these four types of signal in different SNR conditions can be separated by nonlinearity and determination quantified by the proposed method. Therefore, the proposed method seemingly exhibits enough robustness in different SNR conditions. 

## 4. Application to Fault Diagnosis of Gearboxes

### 4.1. Case Study 1

**Figure 3:**
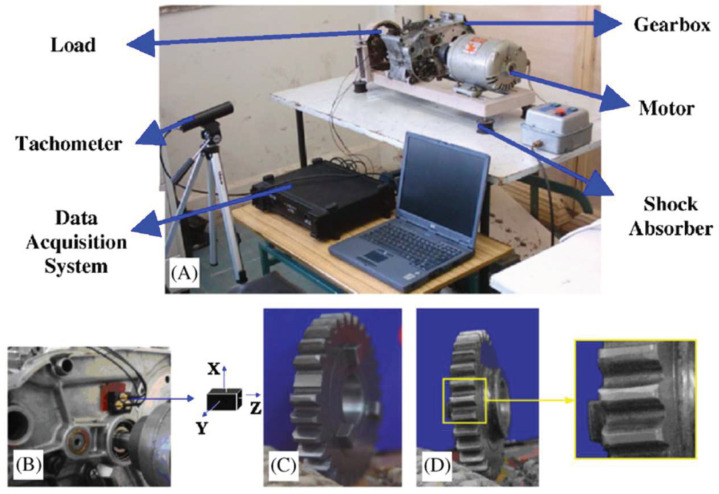
(**A**) A sketch of the first gearbox experiment; (**B**) accelerometer location; (**C**) broken teeth; and (**D**) slight-worn teeth [33].

**Figure 4:**
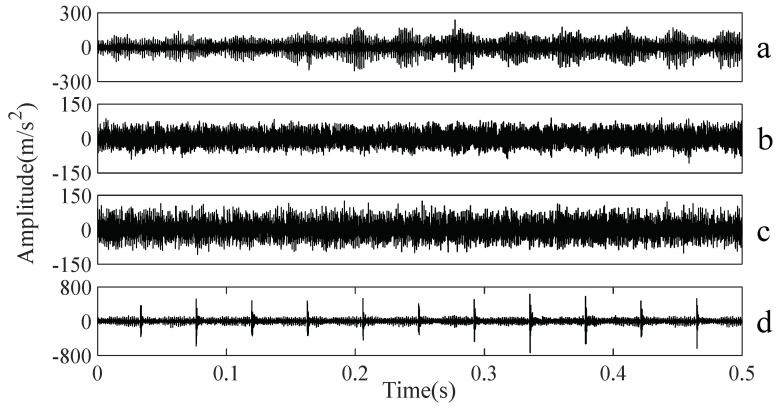
Four types of gearbox vibration data: (**a**–**d**) normal, slight-worn, medium-worn and broken-tooth, respectively.

**Figure 5:**
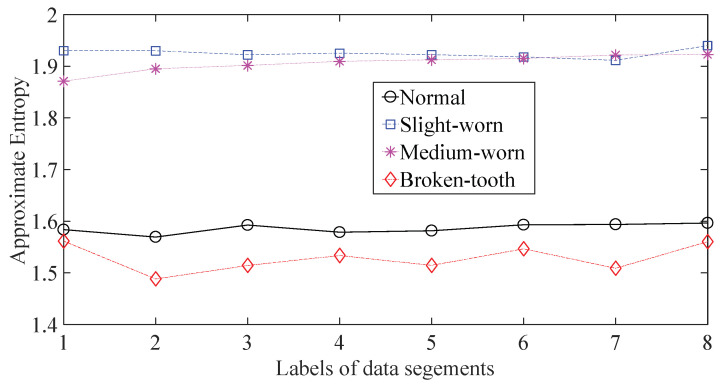
Separations of normal, slight-worn, medium-worn and broken-tooth gearbox conditions by Approximate Entropy.

**Figure 6:**
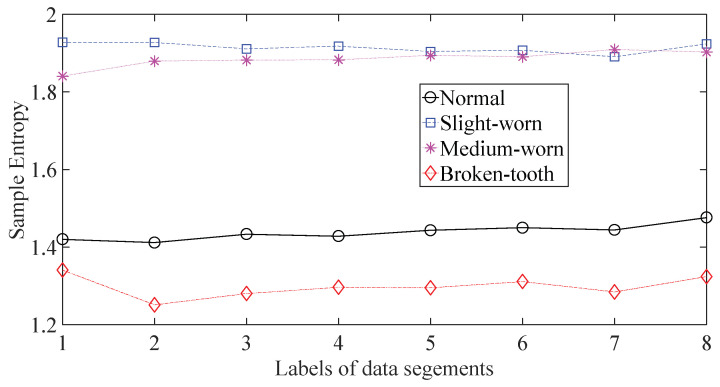
Separations of normal, slight-worn, medium-worn and broken-tooth gearbox conditions by Sample Entropy.

**Figure 7:**
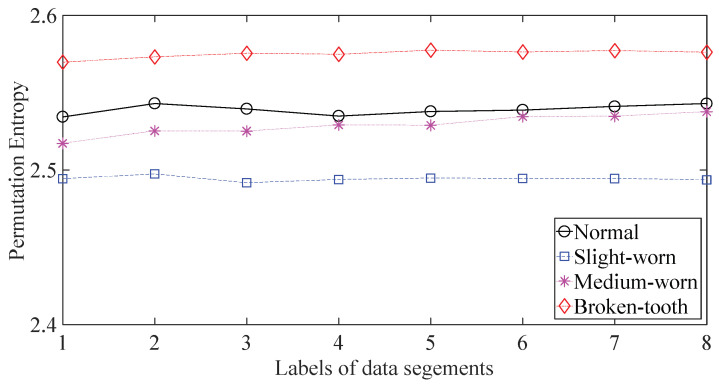
Separations of normal, slight-worn, medium-worn and broken-tooth gearbox conditions by Permutation Entropy.

**Figure 8:**
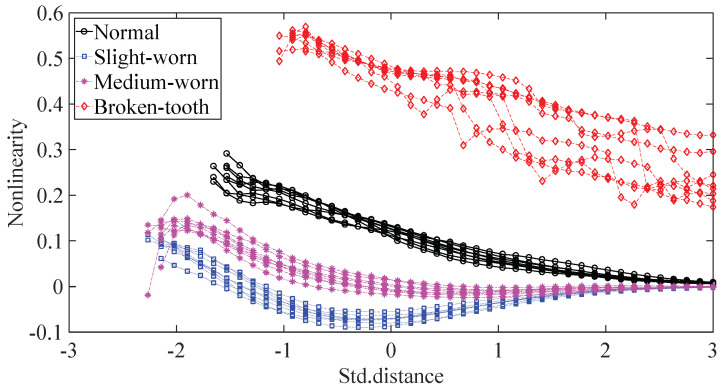
Separations of normal, slight-worn, medium-worn and broken-tooth gearbox conditions by DVV.

**Figure 9:**
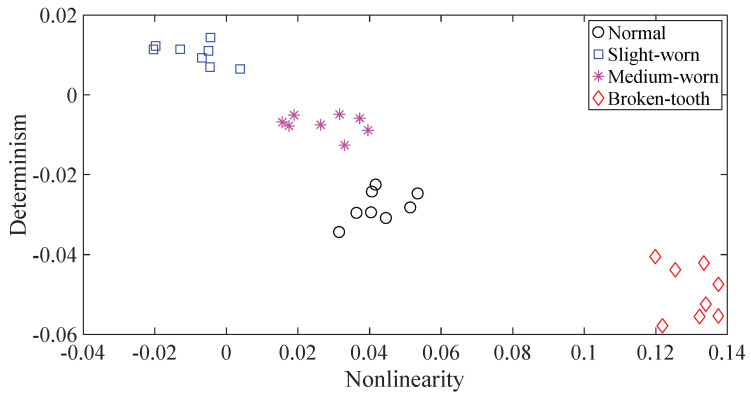
Separations of normal, slight-worn, medium-worn and broken-tooth gearbox conditions by the proposed method.

A gearbox experiment was conducted for evaluating the performance of the proposed method. Vibration data used here were collected from a four-speed motorcycle gearbox. An experimental sketch is given in Figure 3. The experimental rig was supported by four cushion rubbers, which are responsible for cutting off vibrations from the desk in Figure 3. The gearbox was driven by an electrical motor whose nominal rotating speed is 1420 RPM. Four types of gearbox condition were modeled: normal, slight-worn, medium-worn and broken-tooth. Eight pieces of data were collected for each of these four types of gearbox condition, each piece with a sample frequency of 16,384 Hz and a size of 8192. These four types of gearbox vibration data are displayed in Figure 4. It turns out that slight- and medium-worn exhibit similar vibrations and are difficult to discriminate. Consequently, separations of these two similar gearbox conditions can serve as an investigated subject to measure performance of different methods for feature extraction. To begin with, Approximate Entropy showed its application in studying these gearbox vibration data and the results are displayed in Figure 5. As displayed in Figure 5, Approximate Entropy demonstrates few capabilities for distinguishing between slight- and medium-worn (for either of Approximate Entropy and Sample Entropy, the embedded dimension was set as 2 and the tolerance as 0.2 times the standard deviation of the original data). Then, Sample Entropy was applied to probe these gearbox vibration data and the results are shown in Figure 6. A comparison between Figure 5 and Figure 6 indicates that Sample Entropy, whose performance resembles that of Approximate Entropy, also displays little feasibility for separating these two similar gearbox conditions. Afterwards, Permutation Entropy was employed for gaining insight into nature of these gearbox vibration data and the results are reflected in Figure 7 (for Permutation Entropy, the permutation order was set as 3 and the time lag as 1). As reflected in Figure 7, Permutation Entropy fails to clearly differentiate between normal and medium-worn gearbox conditions. Next, DVV was exploited to examining these gearbox vibration data and the results are shown in Figure 8. As shown in Figure 8, the curves for slight- and medium-worn intersect in left ends of these curves. Consequently, DVV seems hard to correctly discover dynamics of these gearbox vibration data. In the end, the proposed method in this paper was adopted for exploring these gearbox vibration data and the results are depicted in Figure 9. As depicted in Figure 9, the proposed method can clearly distinguish between these four types of gearbox conditions. 

### 4.2. Case Study 2

**Figure 10:**
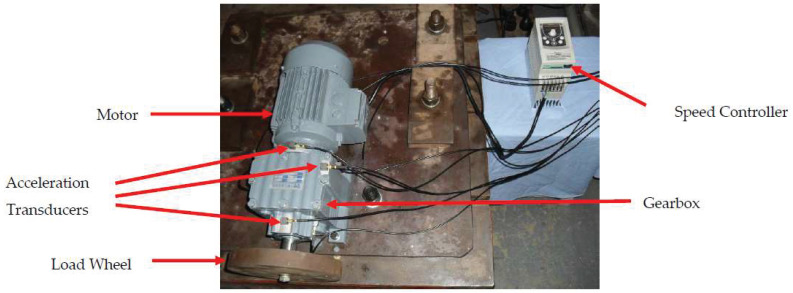
A sketch of the second gearbox experiment.

**Figure 11:**
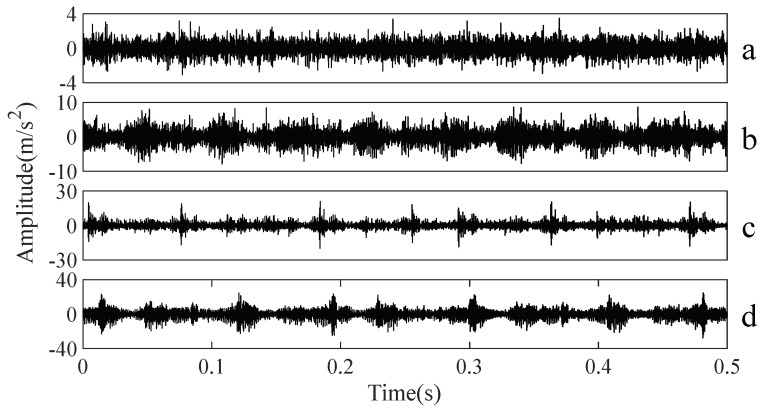
Four types of gearbox vibration data: (**a**–**d**) normal, slight-scratch, medium-scratch and severe-scratch, respectively.

**Figure 12:**
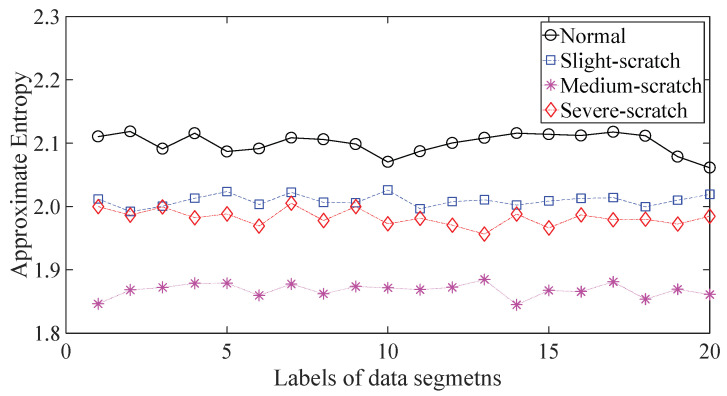
Separations of normal, slight-scratch, medium-scratch and severe-scratch gearbox conditions by Approximate Entropy.

**Figure 13:**
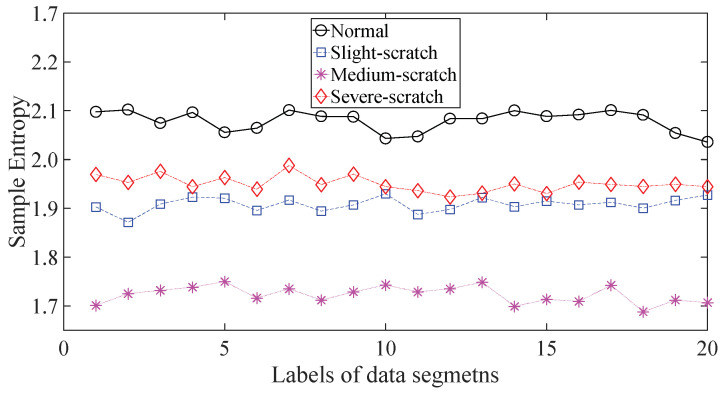
Separations of normal, slight-scratch, medium-scratch and severe-scratch gearbox conditions by Sample Entropy.

**Figure 14:**
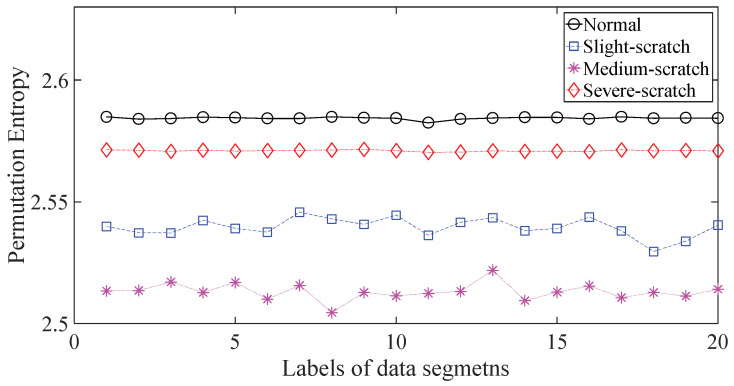
Separations of normal, slight-scratch, medium-scratch and severe-scratch gearbox conditions by Permutation Entropy.

**Figure 15:**
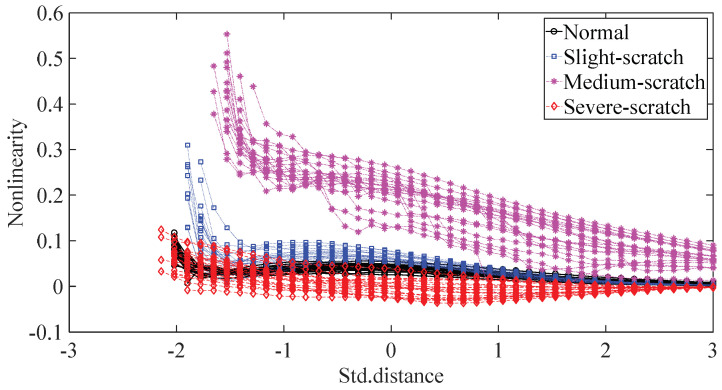
Separations of normal, slight-scratch, medium- scratch and severe-scratch gearbox conditions by DVV.

**Figure 16:**
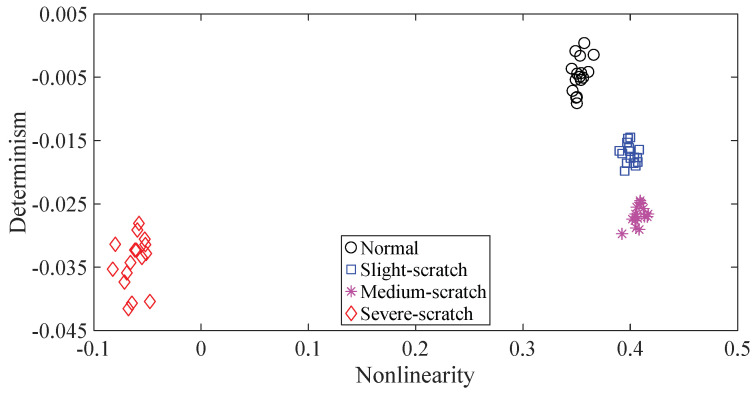
Separations of normal, slight-scratch, medium-scratch and severe-scratch gearbox conditions by the proposed method.

To further assess the performance of the proposed method, another gearbox experiment was carried out. An experimental sketch is depicted in Figure 10. The gearbox used in this subsection belongs to a type of two-stage transmission. The gearbox was driven by a motor governed by a speed controller. Additionally, a wheel was fixed on the output shaft for loading. Four types of gearbox condition were involved in the experiment: normal, slight-scratch, medium-scratch and severe-scratch. Vibration data used in this subsection were gathered from the gearbox case near the input shaft under a rotating speed of 1600 RPM. Twenty pieces of vibration data were collected in each of these four types of gearbox condition, each piece with a sample frequency of 16,384 Hz and a size of 8192. These four types of gearbox vibration data are displayed in Figure 11. First, Approximate Entropy was used to investigate these gearbox vibration data and the results are given in Figure 12. As given in Figure 12, the slight- and severe-scratch are hardly separable. Therefore, Approximate Entropy fails to correctly display dynamics of these gearbox vibration data. Next, Sample Entropy showed its usefulness in analysis of these gearbox vibration data and the results are revealed in Figure 13. As revealed in Figure 13, the slight- and severe-scratch cannot be clearly separated. It follows that Sample Entropy lacks capabilities for distilling essence of these gearbox vibration data. Subsequently, Permutation Entropy was applied to explore these gearbox vibration data and the results are exhibited in Figure 14. As exhibited in Figure 14, Permutation Entropy can serve to distinguish between these four types of gearbox condition and demonstrates superiority over Approximate Entropy and Sample Entropy. Then, DVV was used to undertake an anatomy of these gearbox vibration data and the results are illustrated in Figure 15. As illustrated in Figure 15, the curves for normal, slight-scratch and severe-scratch intersect severely. It means that DVV is unsuccessful in capturing essence of these gearbox vibration data. Finally, the method proposed in this paper was applied to examine these gearbox vibration data and the results are described in Figure 16. Figure 16 points out that the proposed method can clearly separate these four types of gearbox condition. 

## 5. Discussion

The performance of the proposed method was benchmarked against Approximate Entropy, Sample Entropy, Permutation Entropy and DVV by conducting two independent gearbox experiments. In the first experiment, the proposed method outperforms all the other methods. In the second one, the proposed method, comparable to Permutation Entropy, has an advantage over the remaining three methods. In general, the results of these two experiments prove that the proposed method delivers a better performance than the others. As a result, the effectiveness of the proposed method is confirmed in this paper. Furthermore, the capability of the proposed method to process short experimental signals seems to be demonstrated considering the length of experimental signals. 

This paper makes two main contributions. Firstly, this paper introduces a pair of measures for simultaneously describing nonlinearity and determinism of complex data. To achieve this, dynamics hidden in complex data are investigated by making a comparison between generalized Hurst exponents of the shuffle, the surrogate and the original data. Consequently, the deviation of the generalized Hurst exponents of the shuffle data from those of the original data can serve to quantitatively describe determinism of the original data. Similarly, the deviation of the generalized Hurst exponents of the surrogate data from those of the original data can serve to quantitatively describe nonlinearity of the original data. Accordingly, the averages of the deviation are suitable as the measures for quantifying nonlinearity and determinism of data. Secondly, the two-dimensional measure is pioneered in fault diagnosis of gearboxes. In general, with evolution of dynamics of a gearbox, vibrations of the gearbox will become more and more complex. In this manner, vibrations in different running stages of a gearbox display both different nonlinear properties and different noise levels. Consequently, the two-dimensional measure is appropriate for depicting running conditions of a gearbox. 

Although delivering a good performance in fault diagnosis of gearboxes, the method proposed in this paper is still affected by several shortages. First, MFDFA, which is adopted for extracting the generalized Hurst exponent from complex data, needs further refining. Presently, determination of a local trend of data in MFDFA is awkward and time-consuming. In the future, a self-adaptive procedure for determining a local trend of data should be developed. Secondly, a single shuffle or surrogate procedure may cause a systematic error. To eliminate the systematic error, repetitions of a shuffle or surrogate procedure are necessary. However, the repetitive magnitude requires being optimized. This problem will be solved in the future due to a limit of contents of this paper. 

## 6. Conclusions

This paper defines the two-dimensional measure for simultaneously quantifying nonlinearity and determinism of complex data. Then, a novel method for fault diagnosis of gearboxes is proposed based on the two-dimensional measure. Robustness of the proposed method in different SNR conditions was confirmed numerally. Afterwards, the performance of the proposed method was benchmarked against Approximate Entropy, Sample Entropy, Permutation Entropy and DVV by carrying out two independent gearbox experiments. The results show that the proposed method has a clear advantage over the other methods in fault diagnosis of gearboxes. In the future, the proposed method may be expanded to fault diagnosis of other machines and online application.
